# Community-based physical and social activity for older adults with mild frailty: a rapid qualitative study of a collaborative intervention pilot

**DOI:** 10.1186/s12877-024-05604-y

**Published:** 2024-12-19

**Authors:** Jennifer Liddle, Melanie Stowell, Michael Ali, Stephanie Warwick, Alexandra Thompson, Katie Brittain, Adam Brougham, Barbara Hanratty

**Affiliations:** 1 Population Health Sciences Institute, Newcastle University, Newcastle upon Tyne, NE4 5PL UK; 2 Rise, North Tyneside, NE29 6DE UK

**Keywords:** Older adults, Physical activity, Exercise, Frailty, Ageing, Social, Vulnerable, Active ageing

## Abstract

**Background:**

Intervening to modify frailty trajectories may be critical to maintain health and independence in later life. The Active Ageing Programme (AAP) is a 16-week community-based intervention for older people with mild frailty that combines physical activity and social interaction. The programme aims to positively impact resilience and wellbeing, changing the physical, mental and social factors that impact on frailty trajectories. We conducted a rapid qualitative study with the first cohort to understand the acceptability and feasibility of the AAP.

**Methods:**

Purposive sampling was used to identify and recruit staff and participants involved in the AAP for semi-structured interviews. The topic guides covered experiences and perceptions of training and referral, delivery and participation in activities, and benefits and challenges. Data from 20 interviews were analysed using a reflexive thematic approach with inductive coding. The Theoretical Domains Framework prompted consideration of potential individual, social and environmental factors influencing changes in behaviour and practice.

**Results:**

Sixteen AAP staff (10) and participants (6) completed interviews. Two themes were developed: combining motivating components; addressing what matters. The AAP brought together a number of components that supported its acceptability and successful implementation by motivating older people and staff and sustaining their engagement. These included the convenient and familiar location, the opportunity to improve physical health (participants) and to gain experience of an activity intervention, training and confidence (staff), and the variety of activities on offer. The programme was perceived to have potential to improve physical and mental health and increase the personal resources (e.g. knowledge, confidence, motivation) of those taking part. Areas identified as important for the AAP’s future sustainability and success were: tailoring recruitment practices; enhancing organisational communication; and strengthening support for participants to achieve long-term increased physical and social activity and resilience.

**Conclusions:**

Our findings suggest that community-based physical and social activity interventions targeting frailty may be acceptable, feasible and useful. Further work is needed to investigate the impact of such programmes on health and service utilisation. Efforts should be targeted at improving the potential for long-term sustainability of programmes and their impacts.

**Supplementary Information:**

The online version contains supplementary material available at 10.1186/s12877-024-05604-y.

## Background

Global trends in population ageing are increasing the overall prevalence of morbidity and disability in older populations [1, 2]. Between 2017 and 2040 the number of people in the UK aged 85 and over is projected to almost double from 1.4 to 2.7 million [3]. While life expectancy has increased, healthy life expectancy has not [4]. It is estimated that up to half of people over 85 years could be clinically defined as frail [5]. Frailty describes a situation where physical and mental resilience is reduced, resulting in a clinically recognisable state of increased vulnerability [6, 7]. Routine identification of frailty in older people is recommended in general practice in England [8]. 

The development of frailty leads to increased risks of disability and likelihood of falls, admission to hospital, admission to long-term care and death [5, 9]. However, frailty is not inevitable or constant; an individual’s frailty state can improve or deteriorate [10]. Optimising health and independence in later life requires avoiding or delaying the development of frailty [10, 11]. Identification of individuals living with ‘mild’ frailty therefore represents an opportunity to change health trajectories [12]. There is a need to understand how interventions might act at this stage to mitigate or reverse the risk of progression to moderate or severe frailty.

Physical activity can improve the mobility and independence of older people through its positive effects on cognitive and physical functioning and psychological wellbeing [13]. Multicomponent exercise interventions that include resistance, aerobic, balance and flexibility training seem particularly promising to counteract frailty [14]. However, long-term impacts have been more challenging to demonstrate [15, 16]. Moreover, gaps in research regarding the acceptability of activity programmes among older adults in low socioeconomic settings have been documented [17]. Depression and living alone may also contribute to poor acceptance of an intervention [18]. Group-based interventions may improve adherence and commitment to physical activity programmes, offering opportunities to socialise, give and receive support and build feelings of belonging and safety [16]. The incorporation of social dimensions such as these is therefore important, particularly when considering the link between frailty and loneliness [19–22] and the potential for ‘social frailty’ ((threat of) insufficient social resources, social behaviours and attitudes, self-management abilities and general resources to fulfil basic social needs) [23] to worsen frailty trajectories [24]. 

Social prescribing recognises primary determinants of health as social, economic and environmental [25]. Health professionals such as GPs and nurses can refer people to a social prescribing link worker. Social prescribing link workers coproduce personalised care and support plans with people, connecting them to relevant local services run by community partners, including charities and Local Authorities. The Active Ageing Programme (AAP) is a novel place-based collaborative approach which explores the use of proactive, activity-conscious social prescribing, supported by evidence-based physical activity messaging, embedded within routine primary care pathways in Newcastle upon Tyne. The terms ‘physical activity’ and ‘exercise’ are often used interchangeably [26]. In this paper, we use the term physical activity in line with the language used within AAP to capture its exercise, social, training and behaviour change components, while avoiding terminology of ‘exercise’ or ‘sport’ that may be off putting to people who are not active or have barriers to movement. The AAP is facilitated by Rise, a regional charity, funded and supported by Sport England and a number of other local and national organisations [27]. Rise is part of a network of 42 Active Partnerships across the country, working to make physical activity easier and more appealing to people. The AAP targets older adults living in areas of higher deprivation, thus at potential greater risk for poor health outcomes [28], and is delivered by local voluntary and community sector partners.

Patients are proactively identified using electronic patient record data (from EMIS and SystmOne clinical systems) and contacted using the iPLATO messaging tool. Eligible patients are aged ≥ 55 years, coded in records as mildly frail (Electronic Frailty Index [29] 0.12–0.24 and/or Clinical Frailty Scale [30] groups 3–5) and living independently. Patients receiving palliative care, with a recent cancer diagnosis or with severe mental illness are excluded. A health and safety triaging process is in place to identify and exclude patients who have experienced chest pain at rest, lost balance due to dizziness or blackout, received GP advice to avoid light activity, or where there are other reasons why light physical activity should not be undertaken. The AAP is supported by patient-friendly information materials and digital templates for initial patient conversations and referral based on content from Moving Medicine consultation guides [31], embedded within existing health pathways. Example patient information is included as supplementary material Core AAP staff have backgrounds in community health improvement. Activity leads had first aid and cardiopulmonary resuscitation (CPR) training, and a minimum of Level 3 Register of Exercise Professionals (now the Chartered Institute for the Management of Sport and Physical Activity) Exercise Referral qualification. Staff involved in behaviour change activities had relevant behaviour change and motivational interviewing qualifications and experience. In addition, a workforce development offer to operationalise the concept of ‘activity-conscious’ social prescribing was made available to social prescribing link workers, wellbeing coaches, activity service providers, and health care professionals associated with the primary care network. The aim of the training is to improve shared knowledge, skills, and confidence to deliver brief physical activity messaging and interventions in line with government guidance [32], as part of ongoing care and community support.

Participants are supported to improve physical ability and resilience over a 16-week period. Improving resilience within AAP is conceptualised as equipping and preparing participants to better deal with day-to-day physical and social activities, situations and events, including those that may be difficult or stressful. A close parallel is Pooley and Cohen’s definition of resilience as ‘the potential to exhibit resourcefulness by using available internal and external recourses in response to different contextual and developmental challenges’ [33]. Through improving willingness to interact socially with others and motivating participants to take ownership of their health and continue improving, the programme aims to reduce the likelihood of worsening health and related dependence on health and care services [34]. The intervention involves weekly two-hour structured physical and social activity sessions delivered at a community ‘hub’ and facilitated by an activities lead. Following an hour of physical activity, each session concludes with a social hour including refreshments. The activity timetable includes a range of indoor activities including yoga, bowls, tai chi and circuit training, with one activity programmed each week on a rotating basis. Some social hours also involve an educational or behaviour change component, where educators present topics such as healthy eating, increasing daily activity, goal-setting, managing long-term conditions, and maintaining mental wellbeing. Local activity opportunities are identified, collated and made available as an A-Z directory with the aim of highlighting opportunities for participants to supplement weekly AAP activities and maintain increased activity levels beyond the end of the programme.

For interventions to achieve impact in improving older people’s frailty trajectories, it is vital to understand the characteristics of interventions, staff and participants that promote participant engagement. The aim of this qualitative study was to understand participant and staff perceptions and experiences of the AAP during its pilot phase.

## Methods

This work is reported in accordance with the Consolidated Criteria for Reporting Qualitative Research (COREQ) Checklist [35]. 

We undertook a rapid qualitative study of the AAP pilot (first cohort of participants) which was implemented in one Primary Care Network (PCN) across its three GP practices. With capacity for 15, the AAP aimed to recruit a first cohort of 10 participants. Adopting a phenomenological approach, the purpose of our study was to: understand how the intervention was experienced by participants, referral and delivery staff; and to provide insights into the acceptability of the intervention, feasibility of implementation and the perceived benefits and challenges.

This study was conducted in July – November 2022. The project received ethical approval from Newcastle University Research Ethics Committee (REC) (Ref. 21499 − 2022). The intervention is ongoing in October 2024, with 22 cohorts completed across 24 GP practices.

### Study procedures

**Fig. 1:**
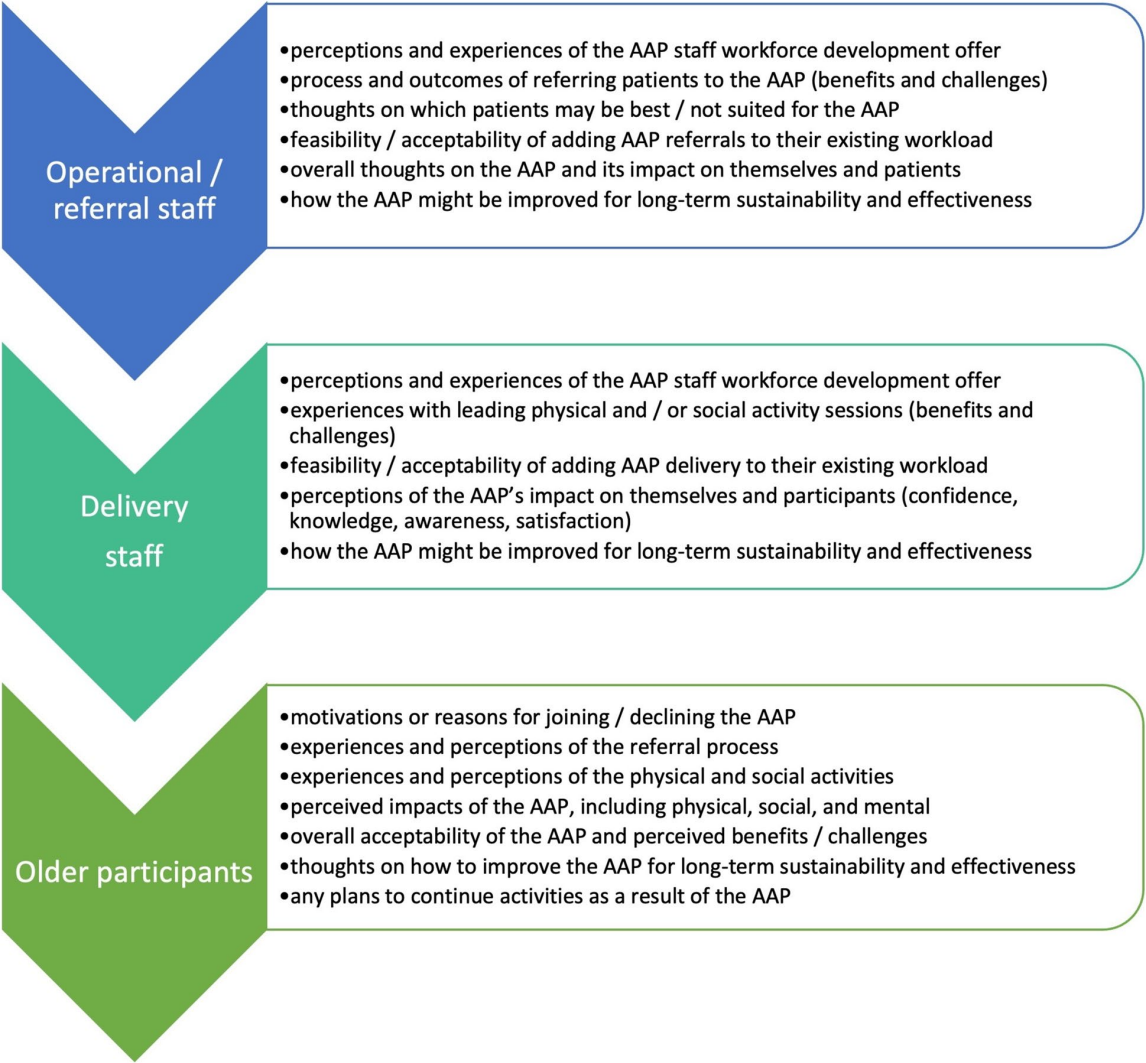
AAP interview topics by respondent type

A semi-structured interview guide was developed for each category of respondent (AAP operational/referral staff, delivery staff, or programme participant) in consultation with clinical experts and programme operational leadership (MA, AB). Interview topics were designed to elicit experiences and perceptions of the most relevant aspects of the programme for each respondent (Fig. 1). The study team also sought feedback from older members of an existing patient and public involvement (PPI) network [36] about the questions designed for AAP participants, to ensure their clarity and relevance, and to identify any omissions.

Recruitment began midway through the 16-week AAP pilot. Purposive sampling, was used to recruit staff interview participants within categories of activity delivery/non-delivery, and role type. All AAP participants in cohort one were invited to participate, with no exclusion criteria other than the ability to provide informed consent. Staff interviewees were identified via Rise; AAP participants from the first cohort were identified via the activities lead. In total, 12 staff were directly involved in AAP with an additional 3–4 indirectly involved within the practice settings. There were 9 AAP participants in cohort one. One participant was below the age of 55 but was accepted onto the programme after an ad hoc request from a GP concerned about the individual’s physical deterioration. The research team introduced the study to prospective staff interviewees by email with an information sheet about the study aims. AAP participants were recruited in-person by a researcher at the physical activity sessions taking place in the community hub. The hub was a community Health Resource Centre that provides a range of services for wellbeing and health improvement, commissioned by Newcastle City Council. Anyone interested in participating was provided with an information sheet and then contacted by telephone to schedule an interview. We also attempted to contact patients who were recruited to AAP but later declined or discontinued participation before week eight. AAP participants who had completed their interviews at an earlier stage in the programme were invited to complete a brief follow-up interview once the 16-week pilot was complete.

Interviews were conducted by female researchers (MS or SW), once informed consent was obtained. MS is a master’s-level research assistant and SW a master’s-level general practitioner and research fellow with personal and professional interest in physical activity. Both researchers have more than six years of training and experience in qualitative interview methods and/or patient interaction. There were no pre-existing relationships between researchers and participants prior to the study, though reasons for doing the research were explained in information sheets and reiterated verbally during recruitment and consent processes. Staff were interviewed via Microsoft Teams or phone audio over an 11-week period, beginning in week eight of the programme. AAP participant interviews were in-person in a private room at the AAP location or via telephone. Participant interviews were conducted over a five-week period, beginning in week 9 of the programme, with follow-up interviews between one and four weeks after the pilot was complete. Interviews were recorded, transcribed and anonymised. All study data were stored and managed in line with the UK General Data Protection Regulation (UK GDPR) and Data Protection Act 2018. After each interview, the researcher documented notes, key impressions and reflections for discussion with the study team.

### Data analysis

Reflexive thematic analysis methods were employed [37], with each interview transcript coded inductively by AT (female, master’s-level research fellow with 3 years training/experience in qualitative research) using NVivo (Release 1.6.1), a software programme used to manage and support analysis of qualitative data [38]. All transcripts were checked against original recordings for accuracy. Braun and Clarke recommend an emphasis on immersion, creativity, thoughtfulness and insight as key to ensuring quality in reflexive thematic analysis [39]. Thus, the study team met regularly to refine existing codes, discuss additional codes, and develop themes. Codes and themes were organised in a matrix which allowed identification of connections and key findings across the data, with codes and illustrative quotes reported for transparency. MS and SW engaged in reflexive journaling and additional reflexive notes were added by JL and AT to the matrix document during the analysis period. Theme names were chosen carefully and refined throughout the process from first development into writing up. Findings are interpreted and discussed in relation to the Theoretical Domains Framework (TDF), which offers a lens to consider individual motivations and capabilities alongside social and environmental influences on behaviour [40]. Domains of the TDF framework as they relate to AAP interview data are available as supplementary material.

## Results

Twenty interviews were completed, with sixteen AAP staff and older participants. AAP participant respondents (*n* = 6) included all those who completed the AAP pilot (Table 1). Three of the nine AAP participants withdrew before starting, or stopped attending after the first few sessions. AAP participant interviews lasted between 35 and 52 min (initial interviews, *n* = 6) and between 17 and 22 min (follow-up interviews, *n* = 4). Despite multiple attempts, no participants who had declined to participate in the AAP were successfully recruited. AAP staff respondents (*n* = 10) included four delivery staff: one activity lead and one instructor from a community partner organisation, and two self-employed instructors (Table 2). Six operational (or non-delivery) staff included three managerial/administrative staff, two social prescribers, and one clinical director. AAP staff interviews lasted between 21 and 49 min.

**Table 1:** AAP participant respondent characteristics

	AAP participants (*n* = 6)
Age, mean (range)	66 (53–71)
Gender, n (%)	
*Man*	3 (50)
*Woman*	3 (50)
Ethnicity, n (%)	
*White British*	4 (67)
*Asian British*	2 (33)
Marital status, n (%)	
*Divorced*	4 (67)
*Married*	2 (33)
Housing tenure, n (%)	
*Homeowner*	4 (67)
*Renter*	2 (33)
Living arrangements, n (%)	
*Living alone*	3 (50)
*Living with partner / partner and child*	3 (50)
Employment status, n (%)	
*Retired*	5 (83)
*Disabled, not working*	1 (17)

**Table 2:** AAP staff respondent characteristics

	Delivery staff	Non-delivery (operational) staff	Total AAP staff (*n* = 10)
Age, mean (range)	42.5 (23–63)		39 (23–63)
Gender, n (%)			
*Man*	3 (75)	1 (17)	4 (40)
*Woman*	1 (25)	5 (83)	6 (60)
Ethnicity, n (%)			
*White British*	4 (100)	6 (100)	10 (100)
Employment status, n (%)			
*Full-time employed*	4 (100)	4 (67)	8 (80)
*Part-time employed*	0 (0)	2 (33)	2 (20)
Number of years in profession / role, mean (range)	5.75 (0.5–11)	5.1 (0.5–10)	5.4 (0.5–11)

### Interview themes

Two themes were generated from the data: combining motivating components; addressing what matters. Interview themes, codes and illustrative quotes are presented in Table 3 and described below.

**Table 3:** Qualitative interview themes and illustrative quotes

Theme	Code	Illustrative quotes *P* = Participant; S = Staff (D = Delivery, Op = Operational)
Combining motivating components	Improving physical health in a familiar setting	*‘Well*,* the idea is very attractive to me and I do want to partake in these sort of things*,* especially the physical aspect of it*,* but that is more selfish reasons that ****I want to improve my stamina***. *Yeah*,* so it attracted me straightaway.’ – P2* *‘In fact*,* I went along ‘cause ****it’s only five minutes away****’ – P4* *‘****I do a rehab as well***. *I do that twice a week. ****I thought if it’s going to be like that***,*** it’ll be great ****because I really enjoy it…If it gets me out the house*,* yeah*,* that’s fine. It’s good. Try it and you’ve got nothing to lose.’ – P1*
	Opportunity to develop professional skills and job satisfaction	*‘ I was happy to do it yeah. It sounds quite interesting*,* obviously I was given the timetable…I thought it would be quite ****a good experience…this being the first one****…’ – S1(D)* *‘If there’s people there that want to do tai chi*,* I am happy to provide it’ – S8(D)* I: *‘Have you or any of your colleagues that you know of ****completed any training ****as part of the Active Aging Programme?’* S10(Op): *‘I know it was offered up to some of my colleagues*, ***I can’t remember if anybody took it up ****or not.’*
	Manageable workload	*‘…****the referral process is really easy***, *it’s just a very straightforward form which it’s obviously uploaded into our GP system. So it means all the patient information’s already populated. So ****it’s just a case of ticking which programme it is you’re referring somebody to***, *and then a little bit of free text about additional information that the person receiving it needs to know…’ – S7(Op)* *‘It’s definitely ****not as time-consuming as I thought****…I’ve got a set two hours each week to* [facilitate], ***but I am also doing additional hours to do the organising****…but it depends what needs to be done’ – S1(D)*
	Motivating factors	*‘…it was fun…we all enjoyed* [indoor bowls]. *And ****when you have been to a session that you enjoy***, *and you go into the hour-long session afterwards ****people are more vocal because they are in a brighter mood****…there was some laughs and things going on and I think it just lifted the mood a little bit so*,* when we came into the room for the discussion the second hour*,* we were all quite chatty.’ – P6* *‘I think it’s not just about a willingness to be there. I think ****it’s about a willingness to want to move and also a faith in oneself ****to be able to listen to themselves enough to stretch a little*,* keep themselves safe but not go too far.’ – S2(D)* *‘[The activity lead]* dealt with everything…He spoke to me this morning. He checked on me on Friday and he checked on me this morning…**He’s the referee**’ - P1
	Communication issues	*‘She thought she was coming to teach us how to be instructors*,* and then*,* realised that she wasn’t*,* so that session didn’t go very far*,* but she said she would change it in order to be suitable for the next time which was good. I think ****possibly because it was the first one***,*** there’s been a little bit of disorganisation***, *let’s just say*,* or lack of communication*,* which I think the next lot will benefit from.’ – P6* *‘Maybe just a bit more coordination. I don’t have a personal number for the* [instructor who didn’t turn up] *otherwise I would’ve called them myself. So ****maybe get some contact details in case a hiccup like this happens again****…****Sometimes it does get switched and I don’t get notified about it***. *I’ve got to adapt to it.’ – S1(D)*
	Tailoring to individuals	*‘I’m sure if she’d done the same things which she was doing*,* but ****at a slower pace***,*** I might have managed***. *Only two of us found that difficult. The rest managed it*,* but they said it was hard. Sometimes*,* the same thing can be done at a slower rate’ – P1* *‘There were two at the back who probably would have ****struggled with my accent and telling them to do an exercise. ****It was clear that… when they were all on their stations*,* I was moving my arms up and down or I was* [demonstrating] *a chest press so they could follow those actions as well’ – S6(*D) ‘I’d like to think that they would be able to facilitate some smaller **groups for the Czech community**,** the Romanian community**,** Bengali***…*they tend to be the three most common alternative languages I come across. And I feel like **there would be a large enough interest** to refer people specifically to those groups’ – S7(Op)
	Tracking and setting goals	*‘****That’s the drawback ****there. There needs to be some progress* [tracking]*… but then ****these people coming in from the outside haven’t got the time***, *really*,* because in one hour*,* they have to do the thing and they* [can’t] *track each and every person. It’s very difficult.’ – P1* ‘…at the start we’d done a timed “up and go” assessment…And they also did a questionnaire where it asked about the quality of life about like mobility, anxiety, depression. And then **in between we don’t do any midway**,** it’s kind of just like week one to week 16** and then we’ll compare the differences’ *– S1(D)*
Addressing what matters	Increase in personal resources required for being active	*‘I’m ****more motivated now ****so I am…even sometimes when I’m struggling with my arthritis*,* I know I’m coming here and I think*,* “I’ve got to get up.” Whereas*, ***other times***,*** I would have thought***,*** “I’ll just stay and I’ll just not bother****”’ – P5* *‘****They teach you new things***…*Now*,* I do a bit more training at home. I know I don’t need any equipment to do any training at home with the exercises they’ve taught us’ – P2*
	Perceived positive impacts on physical and / or mental wellbeing	*‘Yeah*, ***I feel a lot fitter and I feel like I’ve come back to being myself again ****instead of being trapped. You get trapped inside your body but every week*,* you just seem to open up a little bit more than before’ -– P4* *‘So I think it had like ****a double benefit***,*** you were moving but it lifted your mood***, *do you know what I mean’ – P3* *‘The feedback I had was at the end of the class*,* they said*,* “****That was very calming***, *that was very relaxing and soothing”’ – S8(D)*
	Limited social impacts perceived	*‘****You can’t bring a few people together to bond together just like that***, *just by meeting in a meeting room. It takes more than that’ – P2​* *‘I think that maybe could have been a little bit more structured…maybe the first couple of weeks*,* we should have had somebody like a band master to get it all going*,* maybe ****bring some subjects to be discussed****…’ – P6​*
	Potential of health education sessions recognised	*‘…I live on my own you know*,* you can’t always be bothered to cook for yourself and ****you get into bad eating habits****…maybe you could do a cookery session or maybe you could get people in to give you some ideas of how you could ****budget and have nutritional meals on a small amount of money***…*that would encourage people to want to be part of that’ – P6* *‘I think the group seem to enjoy it.* ***They were asking questions and got involved*** *with it’ – S3(Op)*
	Tailoring recruitment practices	*‘I found out about this*, ***it was actually a bit confusing ****because I got this text message. And ****it looked suspicious ****because it had funny symbols in like hashtag and all funny symbols in and I thought it was a scam*,* so I ignored it the first time’ – P3* *‘I think there is a need for it as well ****within different networks in different parts of the city ****whereas you know*,* working in the east side of the city there isn’t anything necessarily there whereas a lot of things are in the inner west of the city…****people in the east side of the city have literally nothing in terms of exercising***, *especially’ – S9(Op)* *‘I would be ****a bit hesitant about referring somebody***…*if they had poor English speaking skills*,* or they ****didn’t have English as a first language***. *I think that ****could become really***,*** really challenging***, *not just for them*,* but also for the facilitators*…*I would probably be a little bit more ****reluctant to have the conversation ****with them about the programme*…*I’d probably be a little bit ****resistant to refer them****’ – S7(*Op) *‘…we ****need more training ****in what this programme actually is*,* how it’s delivered*, *what it looks like. ****How it would look for a service user we are referring****…because from the resources that we’ve been sent… maybe it’s just me that hasn’t picked it up correctly.’ – S7(*Op)
	Long-term engagement	*‘Instead of offering 16-week courses*,* you can ****do just a four-week course***,*** but three times a year or twice a year. ****So that way*,* you can get more people coming at regular intervals because when you tell them it’s…****16 weeks***,*** that’s four months. A long time***, *but I think if they were done in a slightly concise way’ – P2* *‘When you have got somebody participating in a group*,* that’s a good time to ****signpost them to other places where they can go***. *You know*,* they’re already taking part and probably motivated to do something else as well.’ – P6* *‘I think ****if we’d had a larger group***,*** it would have been a bit better****…in terms of the dropout rate*,* you see it with anything…if it’s a smaller group*,* then you’re not going to have that many there. ****If it’s a social group***,*** you’re wanting a good amount of people***. *I know some groups still do work but I think this would benefit being a little bit larger.’ – S3(Op)*

#### Combining motivating components

This theme captures the idea that the AAP brought together a number of components that support its acceptability and successful implementation by motivating older people and staff and sustaining their engagement throughout the pilot period.

Among AAP participants, the opportunity to improve their physical health was the primary reason for joining the programme, as opposed to the opportunity to socialise or generally improve fitness. All participants described having physical conditions which they felt could be improved by the programme. These included overweight, diabetes, chronic obstructive pulmonary disease (COPD), rheumatoid arthritis and mobility issues. Whilst several participants acknowledged that starting a new activity can be intimidating, none expressed any hesitation about joining. There were other factors that older people described as supporting to their decision to join, primarily the convenience and familiarity of the AAP delivery location. A minority also had experience of similar physical activity programmes previously or concurrently, such as pulmonary rehabilitation programmes, which provided a sense of what to expect from the AAP.

Among staff, the AAP was viewed positively and described as an opportunity to gain experience, training, and confidence in supporting older people’s health. Staff generally believed in the importance of remaining active in later life and this was a motivating factor in getting involved. Senior staff were unaware of the workforce training uptake, but several suggested that engagement was not extensive. Whilst the workforce development was not mandatory, staff also described receiving some training/education in relation to delivering the AAP, such as data collection and the benefits of physical activity in later life. However, it was suggested that onsite managerial support on AAP session days might be helpful, as several unexpected issues had already occurred that required delivery staff to problem-solve on their own. Delivery staff also noted that a back-up plan for last-minute staff absences and up-to-date contact information for all external instructors, would be beneficial in addressing on-the-day procedural challenges. Nearly all participants discussed organisational challenges in the programme’s early weeks, such as a delayed start and miscommunications with scheduled facilitators. Overall, staff noted that the workload associated with referring or delivering the AAP was not overly demanding, which motivated them to remain engaged. However, it is worth noting that our data collection occurred at an early stage in the programme and a relatively small number of older patients had been referred or enrolled.

Once taking part in the programme, participants described individual-, staff- and programme-level factors that impacted their motivation to continue. Individual-level factors included internal motivation and desire to engage in new activities, and enjoying structure and getting out of the house. However, participants also described experiencing barriers to participating in certain activities, such as fear of hurting themselves. Staff-level factors included the involvement of delivery staff who were welcoming, supportive and encouraging, and demonstrated relevant knowledge and experience. Having one member of staff (the activity lead) to oversee and serve as a key point of contact was particularly valued. Programme-level factors included: its flexibility if, for instance, a participant needed to leave early or miss a session due to other commitments; the variety and content of physical activities on offer; the interaction, humour and healthy competition involved in some of the (team-based) physical activities, particularly indoor bowls; the welcoming and familiar environment of the AAP location – also previously noted as supporting initial decisions to join the programme. In contrast, lack of enjoyment of a specific activity/instructor’s style negatively impacted motivation. Whilst most participants indicated that the early afternoon timeframe worked well for them, particularly those who experienced pain and stiffness in the mornings, a minority expressed the opposite. Several also noted the logistical challenge of participating in more than one activity programme concurrently.

A range of communication issues impacted both staff and participant experiences. AAP participants frequently voiced a desire for improved communication about what to expect during the programme. This included notifying participants about changes to upcoming activities so they could be mentally prepared, as well as providing take-home information about what they had just learnt. Delivery staff also desired more information about the AAP overall, so they could better understand the dynamics and experience level of the participants.

Whilst it was noted that modification options were often provided, such as an option to complete an exercise seated or standing, several AAP participants felt that certain activities were too physically strenuous for them. Participants felt that better tailoring to individuals could be achieved and that not all external instructors welcomed this feedback. They desired regular opportunities throughout the programme to feedback about how the physical and educational sessions were going, with the aspiration that AAP leadership could responsively adapt subsequent sessions. Similarly, delivery staff recognised the value of collecting participant feedback and adapting when necessary. One staff member suggested systematically collecting feedback midway through a cohort, once all activities and educational topics had been covered at least once, so that participants’ preferred activities and topics could be prioritised in the remaining sessions. Several delivery staff noted the importance of considering participants’ needs around instructor speaking volume, accent, cultural considerations and language barriers.

AAP participants also desired accessible tracking of their progress throughout the programme. Whilst some physical and psychological measures were taken at the start and end of each cohort, participants wished for more opportunities to monitor their own progress to promote individualised goal setting. One participant described how his pulmonary rehabilitation programme did this by having participants track milestones such as the number of wall push-ups they could complete each week. The variety of AAP activities with different instructors, whilst welcomed and appreciated by participants, was recognised as a barrier to observing such tangible changes.

#### Addressing what matters

The second theme conveys the idea that the AAP was living up to expectations. The programme was perceived to have a range of actual or potential positive impacts on participants’ resilience and wellbeing, in line with its aims to change physical, mental and social factors that impact on frailty trajectories.

All AAP participant interviewees reported increased motivation, skills, and confidence to continue being physically active as a result of the programme. Through this increase in personal resources required for being active, the AAP and the variety of activities on offer were described as a gateway to further activity. Most AAP participants also described the programme as being beneficial to their physical health, even if improvements were difficult to quantify. They noted the programme’s specific benefits to their mental or emotional wellbeing described, for example, as uplifted mood, with all reporting that it gave them something to look forward to each week.

In contrast to the overall positive views on the physical activity portions of the AAP, participants were more frequently apathetic about the social hours that followed, describing limited social impacts or benefits. Reported weaknesses of the social hour included its lack of structure, with participants finding it difficult to engage in satisfying conversations with a group of strangers. However, several participants did cite new social connections from the programme that they predicted would last beyond the end of the programme. AAP participants overall found some benefit to the health education sessions that were occasionally embedded in the social hours, with most describing them as informative and useful. However, while the potential of health education sessions was recognised, most participants described room for improvement. Suggested incentives to keep participants engaged in the social and health education sessions included providing snacks, topics for discussion and take-home resources, such as information on how to cook and shop healthily on a budget. Participants also suggested making the educational sessions more engaging and allowing participants to request topics of most interest to them. Staff noted that larger AAP group sizes could improve opportunities for connection among participants.

Analysis identified two topics of particular importance to the AAP’s future sustainability and success. The first was a perceived need to address existing referral and engagement challenges to maintain feasibility and ensure participation of individuals most likely to benefit from the AAP. Tailoring recruitment practices was important from participant and staff perspectives. Nearly all AAP participants discussed some confusion or suspicion regarding the initial recruitment text they received, which came from an unknown number and provided little explanatory information. Messages did not display correctly on some mobile phones, leading to indecipherable content. Several participants contacted their general practitioner’s (GP) office directly for confirmation of the text’s legitimacy. Most participants said they would prefer alternative methods of recruitment, such as a mailed letter or introduction by their GP. Referral staff also described challenges with the referral process, including difficulties establishing the right patient criteria and a lack of information about the programme. Several staff also questioned who is best placed to refer, with some expressing that nurses and social prescribers may be ideal for identifying older patients at risk of isolation, but others noting that referral from a GP may be more persuasive to patients. Referral staff responses aligned regarding the idea that more effort should be put into recruiting populations at greatest risk of frailty and isolation, with suggestions including collaboration with representatives of under-represented groups. More detail about the AAP’s adaptations for all ability levels was also suggested to alleviate any hesitation among patients. However, several staff noted that the AAP’s lack of interpretation services meant that non-English speaking populations would be ineligible, and expressed hesitation about referring otherwise eligible patients.

The second topic related to the AAP’s longer-term sustainability and effectiveness concerned participants’ long-term engagement and activity levels, both throughout the 16-week programme and beyond. Numbers in the first cohort of the programme were relatively small. To improve uptake and retention, participants suggested a shorter programme duration would potentially be less daunting, with the option for individuals to repeat the programme several times a year. Staff also believed that a larger group would improve connection, thereby reducing drop-out rates. AAP participants expressed a desire for more support to continue activities after they completed the full programme, such as information on where to continue indoor bowls or tai chi classes. They did not mention at any time point having received a directory of local opportunities, as had been outlined in the programme’s protocol, suggesting the directory was either not provided or not recognised/remembered as such. At follow-up, participants reported they had been invited to the second AAP cohort, which they accepted. Though it was in response to a direct offer, re-enrolling in the second cohort is indicative that their intentions to remain active had translated into tangible commitments.

## Discussion

This study aimed to explore the experiences and perceptions of older participants and staff involved in the AAP, with particular focus on the acceptability of the programme, its implementation, and its perceived benefits and challenges. The overall perception of the AAP was positive from the viewpoint of both older participants and staff. The programme was viewed as beneficial in its ability and potential to improve physical and mental health and was credited with increasing personal resources of those taking part. The programme was experienced as enjoyable and varied in composition, with only a few less desirable elements highlighted. Both staff members and participants viewed the AAP as being acceptable and feasible to continue, with the majority of staff members indicating minimal burden to their current roles. Findings from this study were fed back to AAP programme leads to support and inform programme development and implementation in subsequent cohorts and wider roll-out.

Mapping findings to the TDF [40] identifies key aspects of behaviour or practice where the pilot was perceived to have made impact or facilitated growth, and highlights those where there were barriers, less change, or little evidence of change. In particular, participants’ social roles, goals and resources remained relatively unchanged by the AAP, with none indicating that the social sessions had impacted their confidence or skills necessary to build or sustain meaningful relationships. Unlike the other curated and targeted components, this social component of the intervention was unstructured with intended pathways to impact unclear, suggesting its presence, composition and purpose require further consideration. However, social influences occurred in other ways; team-based activities such as indoor bowls or circuit bingo had the dual effect of providing an enjoyable form of exercise and a mechanism for participants to bond, compete and laugh with one another. Our finding that this combination was motivating to participants has been noted elsewhere [41, 42]. While it appears that activities generating positive interaction and competition are important in programme engagement, more work is needed to understand how these short-term social experiences might be cultivated and exploited by participants and programme facilitators to impact on social resilience.

Tailoring the programme to individual expectations and abilities was highlighted as important by AAP staff and participants, supporting existing research which suggests that developing personalised, realistic activity goals results in increased physical activity [43]. Previous literature has similarly noted the importance of adapting activities to the specific ability of individuals by adjusting the frequency and intensity of an exercise [44]. These alterations can encourage engagement and prevent participants from feeling like they are holding others back [45]. There was also a common sentiment among respondents that the AAP could do more to support individuals’ long-term goals. Frailty is a dynamic process and benefits gained from physical activity may not last in the longer term if activity levels subsequently decline [46, 47]. In proactively tailoring, interventions may more effectively develop shared perspectives among staff and participants about their purpose, implementation strategies and target outcomes. In particular, ensuring that accurate, appropriate and realistic concepts of active ageing [48, 49] and frailty pervade all elements of programmes like the AAP would be beneficial, incorporating time to sensitively counter ageist or stereotypical beliefs of participants or staff that might influence expectations, concerns about safety, activity levels and goals [50]. This might be especially important in disadvantaged groups, where external expectations around physical fitness and healthy lifestyles may be perceived as threatening rather than as challenges that motivate [51, 52]. 

Whilst not an issue raised by the first cohort of AAP participants, tailoring of the programme was also discussed by staff in relation to cultural considerations and practical concerns such as speaking volume, accent and language spoken by instructors. These topics have been highlighted in previous literature which discusses the importance of offering culturally tailored classes [53]. Given that all delivery staff in our interviews were of White British descent, with an average age of 39, prioritising the recruitment of a diverse workforce may help to facilitate participant engagement among underrepresented groups in programmes like the AAP. However, the question of appropriate cultural tailoring in health promotion interventions is complex, particularly among individuals who are members of multiple minority groups [54, 55]. As this is an area of the literature in which more evidence is needed, careful consideration in practice is therefore warranted.

Interviews with staff and participants revealed that the AAP’s greatest areas for improvement were primarily related to the programme’s environmental context, structures and processes. Surprisingly, these aspects appear to have been elicited rarely in previous literature relating to physical activity-focussed frailty interventions. However, similar factors have been noted in literature relating to general physical activity interventions, highlighting the importance of effective communication across levels of the organisation to avoid the growth of avoidable procedural and structural challenges [56]. Our findings suggest that improvements in engagement and long-term sustainability of the AAP and similar programmes could be achieved by considering ways to improve inter- and intra-organisational communication and cohesion, including enhancing the contribution of delivery staff to decision-making at a strategic level. Activity leads did not have the experience or strategic oversight of AAP management staff, but operated in relative isolation from management staff when attempting to resolve on-the-day procedural challenges, missing opportunities to develop standard procedures for handling and communicating changes to participants. AAP activity instructors, who spent the most time with participants, had ideas and insights that were relevant to the wider programme design and delivery. The varied range of activities delivered by expert instructors was positive for participants. However, the outsourced, peripatetic nature of these instructor roles meant that AAP activity instructors were perhaps the least involved in decisions about programme delivery beyond the content of their individual sessions.

Whilst evidence in support of physical activity interventions for older adults is growing [57], more evidence is needed to understand the mechanisms underpinning long-term sustainability of interventions and physical and social activity levels [58]. Future research may benefit from larger, representative samples and mixed methods to strengthen our understanding of the characteristics and outcomes associated with the most successful community-based interventions targeting frailty. Methodological considerations include: longer-term follow-up periods to assess the physical, social, and psychological impact of these interventions over time; considering the perspectives of individuals who participate in interventions as well as those who decline or withdraw participation; and exploring ways to address identity-related challenges and barriers in these types of programmes. Policy efforts to help older adults to maintain independence in later years could leverage examples of promising interventions such as the AAP, both locally and nationally, to support the reach and impact of ongoing work. An emphasis on targeted approaches could also ensure that programmes with demonstrated effectiveness to prevent frailty are delivered and tailored to the communities that would most benefit from this support.

### Strengths and limitations

This qualitative study offers a comprehensive overview of both staff and participant perspectives of the AAP. We were able to recruit all six of the AAP pilot’s participants and the majority of staff responsible for delivering the programme. Furthermore, we were able to elicit perspectives from staff on both the operational and referral sides of the programme, allowing for an in-depth assessment of where the strengths and room for improvement lie within the programme’s chain of activities. By following up with four AAP participants, we were also able to assess whether perspectives had changed as their time in the programme progressed.

Despite the strong engagement from staff and participants who were already involved, we were unable to recruit older patients who declined to join the AAP. This made it more challenging to understand the reasons why the AAP was not attractive to all patients who were referred, and thus we cannot provide insights into how to improve engagement from this perspective. However, our respondents were open to sharing any hesitations they experienced about the AAP.

As this study was qualitative in nature and involved a small sample size, we were unable to assess differences in experiences across sociodemographic categories such as ethnicity or gender. Frailty and health status may have varied widely within the categories identified as ‘mild’. We did not attempt to collect information on participants’ socioeconomic status. All of the staff we interviewed were White British and thus we did not capture the perspectives of staff from other ethnic groups, if there were any. However, we have identified data that speak to these issues, such as considerations about the language and cultural appropriateness of the AAP for underrepresented groups.

## Conclusions

Understanding the acceptability and feasibility of interventions aiming to change frailty trajectories is important to ensure that individuals can maintain their health and independence in later life. Our findings suggest that, with attention to points raised in this research, the AAP and similar community-based physical and social activity interventions can be acceptable, feasible and useful from the perspectives of both older adults at risk of poor outcomes due to frailty and the members of staff and organisations delivering them.

## Supplementary Information

Supplementary Material 1.

Supplementary Material 2.

## Data Availability

Data that support the findings of this study are available from the corresponding author (JL) on reasonable request. The data are not publicly available due to them containing information that could compromise research participant privacy and consent.
