# Supplementary material for: Community-based physical and social activity for older adults with mild frailty: a rapid qualitative study of a collaborative intervention pilot
Source: BMC Geriatr. 2024 Dec 19;24:1011. doi: 10.1186/s12877-024-05604-y (PMC11657703; doi:10.1186/s12877-024-05604-y)
Supplement: Supplementary file 1 — Supplementary Material 1. [file 12877_2024_5604_MOESM1_ESM.docx]

| Domain | Summary of data – AAP participants | Summary of data – AAP staff |
| --- | --- | --- |
| Knowledge | The AAP has increased or highlighted knowledge / awareness for participants in relation to:   - the benefits of physical exercise - procedural knowledge of how to perform the exercises on offer   Gaps in participant knowledge / awareness included:   - initial information about the AAP during the recruitment process - information about changes to the schedule of activities - specific health topics of interest for the educational sessions (e.g. healthy cooking on a budget) - options for where to continue activities after the AAP is complete | The AAP has increased or highlighted knowledge / awareness for staff in relation to:   - the availability of new community programmes to support older people to remain active physically and socially - how to refer and engage older people into such programmes - how to implement and deliver such programmes   Gaps in staff knowledge included:   - awareness of the AAP and what it involves - awareness of the workforce development offer - procedural awareness, e.g. contact information for external instructors or how to address last-minute scheduling changes |
| Skills | The AAP has increased or highlighted participant skills in relation to:   - skills required to perform new exercises, such as yoga and tai chi   Gaps in participant skills included:   - improved social skills / confidence necessary for growing strong social connections | The AAP has increased or highlighted staff skills in relation to:   - following procedures to refer and collect routine data on programme participants - administration and facilitation of group-based activity programmes with differing levels of participant abilities - implementing programmes with flexibility and adaptability when unforeseen changes or needs arise   Gaps in staff skills included:   - formal training to implement and deliver the AAP and align all staff in meeting its aims |
| Social / professional role and identity | The AAP has strengthened or highlighted participants’ social roles / identities in relation to:   - a minority reported they had developed social connections that could last beyond the programme - participants with long-term conditions felt less ‘trapped’ inside their bodies (P4)   Gaps in changes to participant social roles / identities included:   - an overall lack of feeling that the social hours were producing tangible effects on participants’ social lives and relationships - participants still described physical abilities and limitations in relationship to their age and conditions | The AAP has strengthened or highlighted staff professional roles / identities in relation to:   - providing new roles for staff to promote physical and social activity - building professional confidence in leading groups of older people to promote physical and social activity - affirming organisational commitments to supporting older people’s health and wellbeing   Gaps in changes to staff professional roles / identities included:   - organisational disconnects / lack of cohesion at the referral and training level made it difficult for staff to fully act on meeting the AAP’s aims - organisational disconnects / lack of cohesion at the delivery level made it difficult for staff to fully take the lead when unexpected procedural issues arose - clinical leadership to support and promote staff development and referral uptake |
| Beliefs about capabilities | The AAP has strengthened or highlighted participants’ beliefs in relation to:   - capability to engage in physical exercise independently - capability to improve their physical health and fitness level - capability to fully complete the AAP even if they needed to skip some weeks due to scheduling conflicts   Gaps in participants’ beliefs included:   - capability to complete exercises safely when it felt too physically strenuous | The AAP has strengthened or highlighted staff beliefs in relation to:   - capability to adapt and modify the programme to meet the participants’ needs - capability to carry out AAP responsibilities without it overburdening their existing workload   Gaps in staff beliefs included:   - capability to effectively refer older patients to the programme with full details on what it involves - capability to contact the appropriate people and problem-solve when there were unforeseen scheduling issues |
| Optimism | The AAP has strengthened or highlighted participants’ optimism in relation to:   - optimism that overall, joining the AAP would be beneficial to them   Gaps in participant optimism included:   - negative perceptions of certain delivery staff or activities as being too physically demanding and would not be beneficial to them | The AAP has strengthened or highlighted staff optimism in relation to:   - the benefits of being physically and socially active in later life   Gaps or discrepancies in staff optimism included:   - over-optimism that participants would be open to challenging themselves at certain levels during new exercises - over-optimism that referral staff would be willing and able to understand and refer to the AAP at current levels of oversight / engagement |
| Beliefs about consequences | The AAP has strengthened or highlighted participants’ beliefs about consequences in relation to:   - increased physical activity can have the desired effect on overall physical / mental health and fitness levels   Gaps or discrepancies in participants’ beliefs about consequences included:   - unstructured interactions with strangers are less effective at promoting social connections - lack of ability to track progress can hinder understanding of positive outcomes of participation | The AAP has strengthened or highlighted staff beliefs about consequences in relation to:   - programmes such as the AAP play an important role in keeping older adults active and healthy - programmes such as the AAP can help to engage and teach older people about how to maintain physical and social health in later life - the AAP is feasible and acceptable to both staff and participants   Gaps or discrepancies in staff beliefs about consequences included:   - current AAP referral and engagement methods may be missing key groups who might benefit most from such programmes - language barriers may also prevent key groups from engaging in the AAP - some staff felt that participants experienced psychological barriers which prevented them from challenging themselves physically in a way that would benefit them |
| Reinforcement | Reinforcement for participant engagement and continuation was observed in relation to:   - activities that incorporated teamwork, competition and humour - opportunities to provide feedback on preferred activities and instructors - the structure and routine in participants’ weekly schedules provided by the AAP - welcoming, supportive staff - noticeable improvements to physical and mental stamina   Gaps in reinforcement / disincentives for participants included:   - lack of linkage to other activity opportunities made participants concerned that progress made during the AAP could be lost - lack of support to track progress individually made their progression less obvious and less rewarding - activities or instructors that did not align with participants’ interests or capabilities made them less willing to participate in those activities again - social hours that did not feel supportive to building meaningful connections | Reinforcement for staff engagement and continuation was observed in relation to:   - promoting and serving the aims of the AAP - opportunities to improve professional skills and gain experience - positive interactions and rapport-building with participants - feasibility within existing work responsibilities   Gaps in reinforcement / disincentives for participants included:   - mismatched expectations between staff and participants about the appropriate level of physical challenge - miscommunications which led last-minute scheduling changes and lack of onsite support to manage them - overall lack of information about the AAP and development opportunities |
| Intentions | The AAP has strengthened or highlighted participant intentions in relation to:   - intentions to continue being physically active and to seek out similar programmes in the future   Gaps in participant intentions included:   - AAP participants appeared to already have a high level of motivation to be physically active on joining the programme, so it is difficult to assess how much the programme changed this - the AAP appeared to have less of an impact on participants’ intentions to engage in purely social activities | The AAP has strengthened or highlighted staff intentions in relation to:   - desire to continue working and gaining experience in AAP-related activities and health promotion - improve the programme’s acceptability and effectiveness for participants by taking their suggestions into consideration   Gaps in staff intentions included:   - staff indicated that their intention to refer to the AAP was impacted by a lack of information about patients the programme’s goals and who would be ideal candidates for it - limited time and resource of referral staff also appeared to impact on their intention to prioritise referring patients to the AAP |
| Memory, attention and decision processes | The AAP has strengthened or highlighted participant memory, attention and processes in relation to:   - making the decision to join the AAP and to try new physical activities in a safe environment - learning new skills and exercise movements under the instruction of delivery staff - acquiring new health information to use in their everyday lives   Gaps in participant memory, attention and processes included:   - prior / concurrent experience with similar programmes for some participants makes it difficult to ascertain how much was truly learned in the AAP - feeling overwhelmed by the physical difficulty of certain activities prevented some participants from engaging in them - traumatic memories triggered a minority of participants during the AAP sessions - physical exertion during the first hour left some participants feeling too tired to fully engage in the social hours | The AAP has strengthened or highlighted staff memory, attention and processes in relation to:   - ability to coordinate and lead a group with varied needs and decide on solutions when unexpected logistical challenges arise   Gaps in staff memory, attention and processes included:   - limited time and information about the AAP lessened staff’s memory of the programme and attention toward referral opportunities |
| Environmental context and resources | Environmental context and resources positively impacting on participants included:   - familiarity and convenience of AAP location for ease of access - welcoming environment and encouraging staff - time of day was feasible for majority of participants   Gaps in participant environmental context and resources included:   - some participants felt the AAP ended too late in the day due to other responsibilities - snacks and discussion topics may have helped the social hours to be more inviting - more take-home resources and linkage to other activities were desired - recruitment texts stuck out to participants as suspicious initially - referral / discussion of AAP with health professional before joining would be helpful - some staff / activities were noted to be less receptive to needs for modification | Environmental context and resources positively impacting on staff included:   - some referral staff noted the referral process was relatively simple and straightforward - referral staff appreciated acknowledgement of the time commitment required by administrative staff, not just clinicians   Gaps in staff environmental context and resources included:   - staff noted key older populations may be missed due to lack of support for non-English speakers - referral staff reported lack of physical information / organisational cohesion about the AAP and development offer - staff noted the importance of financial and organisational support for programmes like the AAP to be sustained - staff had mixed perspectives on who / where referral should come from given time and resource constraints |
| Social influences | Positive social influences for participants included:   - healthy competition involved in some physical activities - supportive and friendly staff members - presence of other AAP participants motivated them to keep going during physical activities   Negative social aspects for participants included:   - mismatched expectations between some delivery staff and participants could be a source of tension - educational and social hours were less conducive to rapport and connection-building - some AAP participants had a difficult time finding commonalities / acceptance with peers - several participants were aware that they had more physical limitations than others in the group | Positive social influences for staff included:   - strong rapport between delivery staff and participants   Negative social aspects for staff included:   - tension between some staff and participants who were unhappy with the activity / instruction that day - lack of cohesion among team members led to misunderstandings or disconnects in information |
| Emotion | Positive emotional impacts for participants included:   - overall mood-lifting effects of participating in the AAP - fun and laughter involved with certain activities, e.g. indoor bowls and circuit bingo   Negative emotional aspects for participants included:   - fear or anxiety around hurting oneself or being too physically exhausted from the activities - lack of enjoyment for some participants with certain activities | Positive emotional aspects for staff included:   - positive feelings about delivering the AAP and feasibility of responsibilities taken on - dedication to the AAP’s aims and promoting healthy ageing   Negative emotional aspects for staff included:   - competing demands - fear of public speaking - lack of emotional investment among some staff |
| Behavioural regulation | Facilitators of participant behaviour regulation included:   - increased motivation to maintain routine and attend the AAP when they otherwise would have stayed home - increased motivation to exercise on their own and maintain physical fitness levels - desire / plans to continue seeking and participating in similar programmes in the future   Barriers to participant behaviour regulation included:   - lack of consistent / accessible progress tracking made it difficult for participants to self-monitor and set goals - lack of linkage / take-home resources made it difficult to plan and implement new routines and seek other activities | Facilitators of staff behaviour regulation included:   - opportunities to feedback on the AAP in its early stages has allowed for adaptations to be made - training and skill development opportunities have allowed staff to incorporate the AAP into their career plans / goals   Barriers to staff behaviour regulation included:   - more opportunities to train and develop skills would further promote career planning and progression - more opportunities to feedback about the AAP would promote further adaptations and action planning |
