# Supplementary material for: Community-based physical and social activity for older adults with mild frailty: a rapid qualitative study of a collaborative intervention pilot
Source: BMC Geriatr. 2024 Dec 19;24:1011. doi: 10.1186/s12877-024-05604-y (PMC11657703; doi:10.1186/s12877-024-05604-y)
Supplement: Supplementary file 2 — Supplementary Material 2. [file 12877_2024_5604_MOESM2_ESM.pdf]

Active Ageing 2023

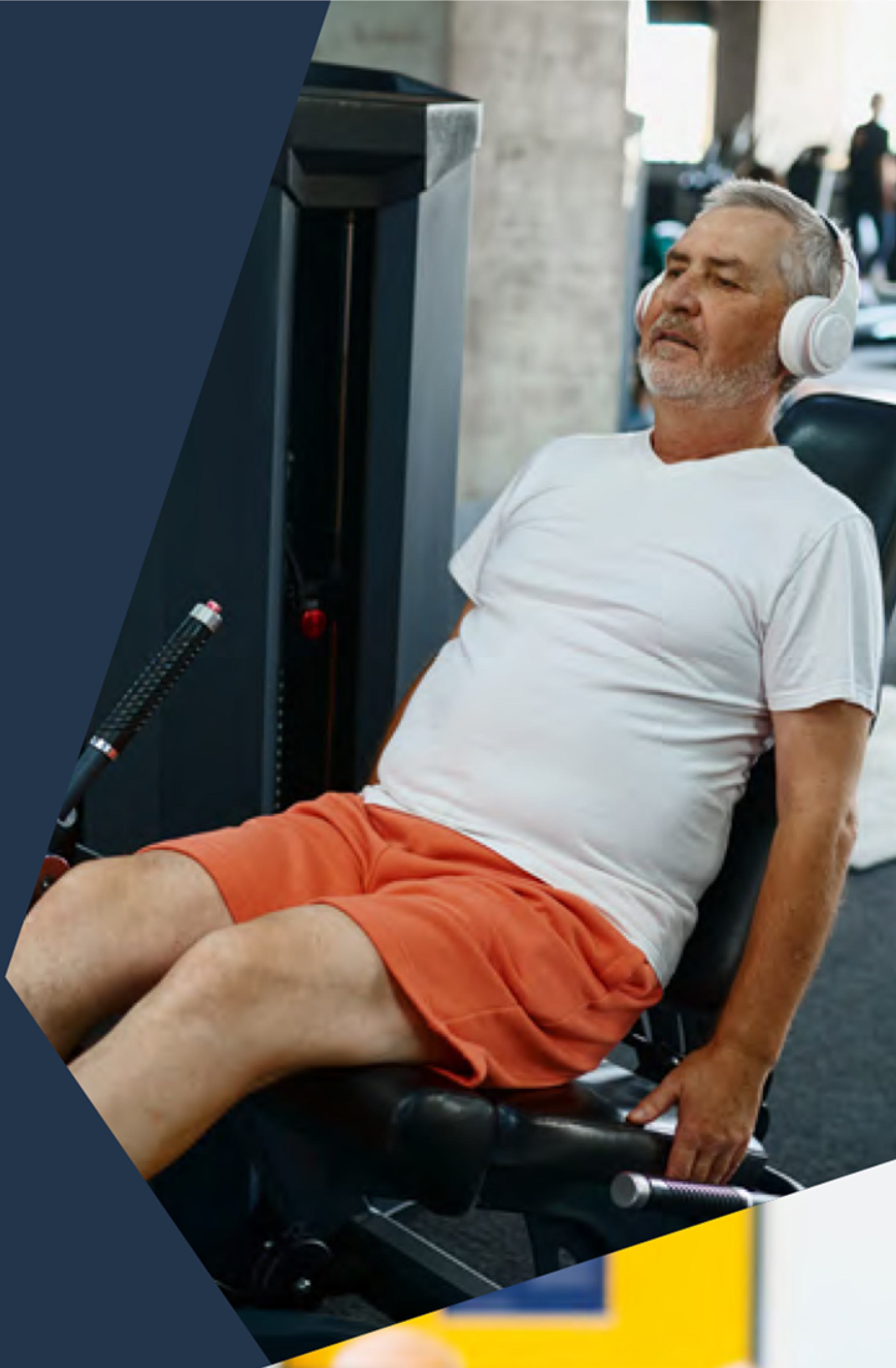

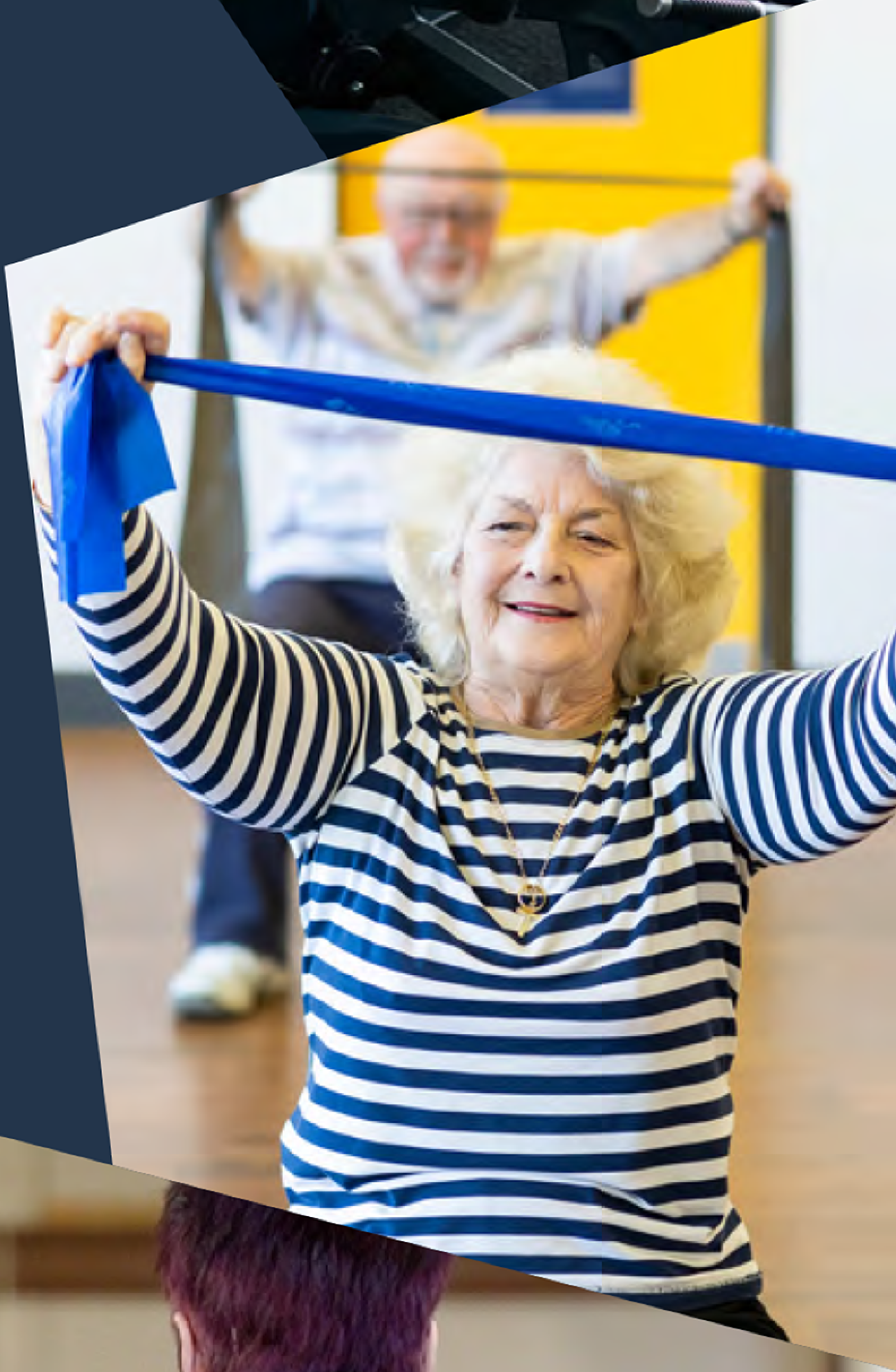

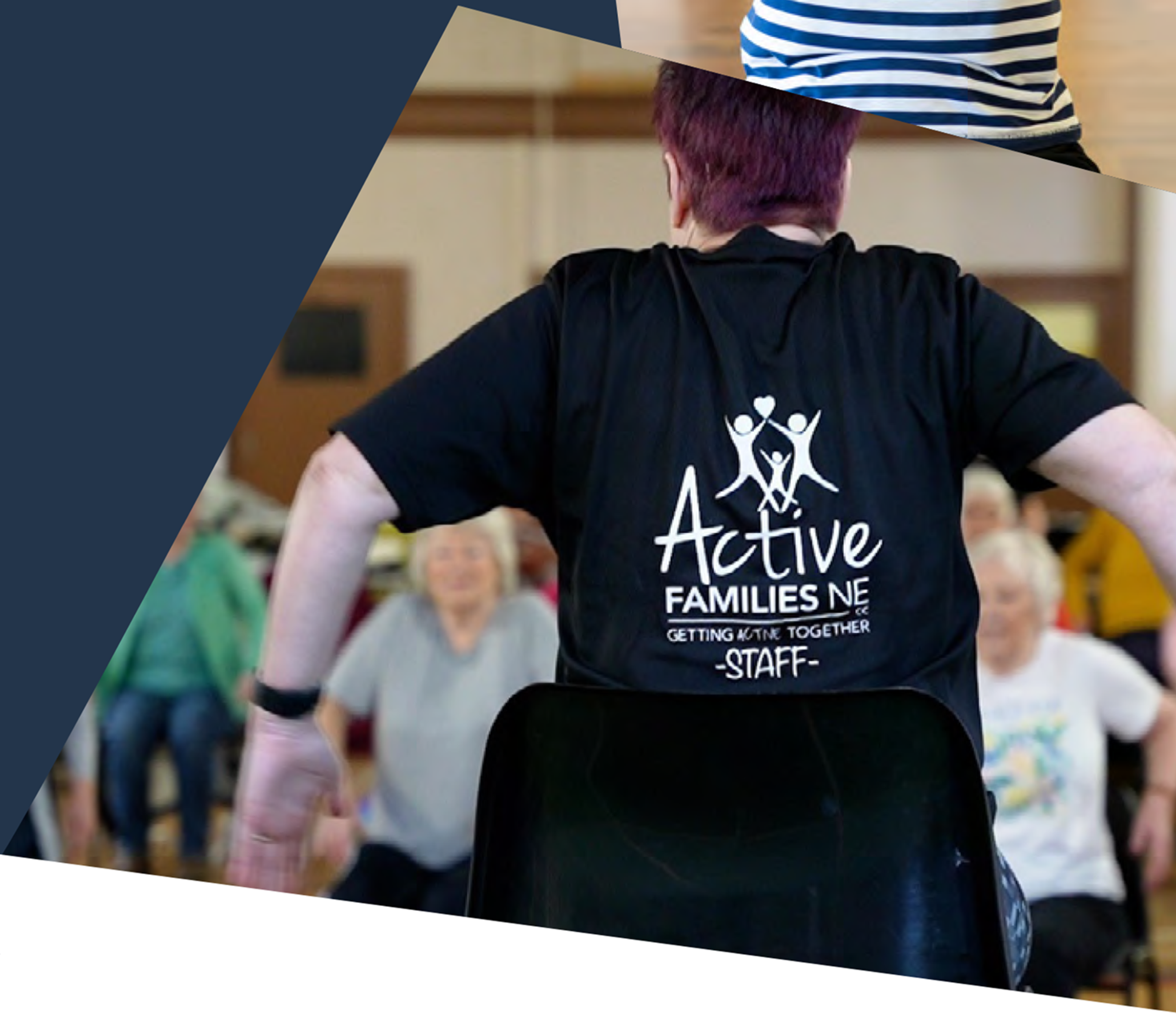

RISE.

Healthworks
the community health charity

GORILLA TRAINING

mindful therapies

Active FAMILIES NE

West End Family Health

BOWLS DEVELOPMENT ALLIANCE.

Would you like to be more active and take control of your health? Join our Active Ageing programme!

Our Active Ageing programme is **FREE to attend** and is delivered by our fantastic Community Health Improvement Team.

Come along to socialise, try fun group-based activities and find out how to improve your health and wellbeing over a cuppa!

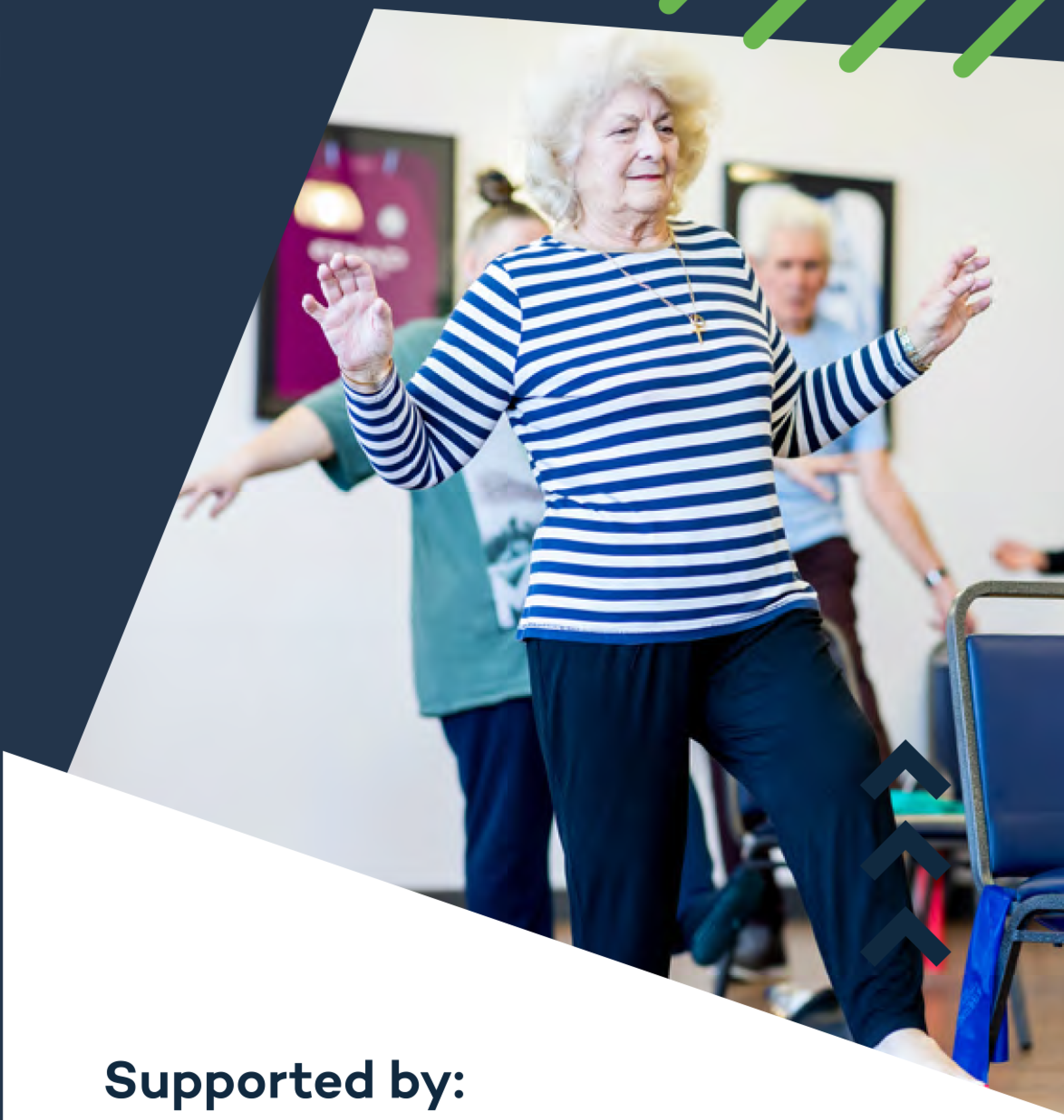

Where?

Healthworks, Adelaide Terrace,
Newcastle Upon Tyne, NE4 8BE

Tuesdays 10:30am to 12pm

For more information,
please contact:

admin.healthworks@nhs.net
0191 272 4244

Supported by:

RISE.

Healthworks
the community health charity

West End
Family Health

BOWLS DEVELOPMENT ALLIANCE.

GORILLA TRAINING

mindful therapies

Active FAMILIES NE

Being active prevents disease and keeps you healthy

What good things could being more active do for you?

- Improve general wellbeing
- Manage stress
- Improves mood
- Maintain healthy weight
- Live longer and better

Reduce your risk by being more active. All adults keeping physically active reduces your chance of:

High Blood Pressure	-50%
Type 2 Diabetes	-40%
Coronary Heart Disease	-40%
Cardiovascular Disease	-35%
Stroke	-30%
Cancer (Breast, Colon, etc)	-25%
Joint and Back Pain	-25%
Falls	-21%
Obesity	-10%

How can being active reduce my stress?

Healthy outlet for nervous energy, distraction from negative thoughts and feelings.

Reduction of fear, symptoms and catastrophisation. Increased sense of calm, better overall wellbeing.

Less stress and anxiety.

Better sleep, better relationships, socialise more.

Follow these top tips to keep you active:

01 If physical activity is new to you, start slowly and build up gradually over three to six months

Build activity into your daily routine – anything you can do to increase your movement will be beneficial. An example might be taking the stairs rather than the lift. 02

03 Some discomfort during and immediately after activity does not mean damage. It is normal for anyone to experience some muscle soreness after doing a new activity. Over time, as your body adapts, this will reduce.

Break up your sitting time – either at home or work. Standing up and taking a break from the screen will give you more energy, improve your concentration and help you be more physically active. 04

05 Find out what is available in your local area – your local council will have a list of activities and groups you could try. If you have a local community care coordinator (who often works closely with your GP surgery) they will have lots of information about what is available.

Choose an activity you enjoy – you are much more likely to stick with it. This may mean trying a variety of activities before you choose one to do regularly. 06

07 Tell your friends & family how you are trying to be more physically active. They may provide important moral support (as changing your habits is not easy) and may also be able to help you make time by helping out with things like childcare.

Consider undertaking physical activity with a friend, or joining a group – this might be more enjoyable, and you can motivate each other. You might also find that this helps you to feel safer, for example if you are walking/exercising outside. 08

09 Be realistic – some days will feel easier than others, and this is normal. Aim to build more movement and less sitting time into your normal life.

Recreation

- Yoga/pilates/ tai chi
- Taking the dog for a walk
- Walking and rambling
- Pool based activity /swimming or aqua class
- Exercise class

At Home

- Gardening
- Housework – doing the vacuuming
- Carrying the shopping home
- Home based exercise

Travelling

- Cycling
- Get off the bus a stop early
- Walk
- Park at a car parking space further away

At Work

- Walking up the stairs
- Walking to a meeting
- Take an active lunch break
- Standing to talk on telephone

Being active is important for falls and frailty

What good things could being more active do for you?

- Reduces risk of falling
- Improves ability to perform daily activities
- Reduces fall-related fractures
- Improves functional ability
- Faster walking speed
- Reduces progression of frailty
- Increased muscle strength
- Better balance

Reduce your risk by being more active. All adults keeping physically active reduces your chance of:

High Blood Pressure	-50%
Type 2 Diabetes	-40%
Coronary Heart Disease	-40%
Cardiovascular Disease	-35%
Stroke	-30%
Cancer (Breast, Colon, etc)	-25%
Joint and Back Pain	-25%
Falls	-21%
Obesity	-10%

How can being active reduce the risks?

You are motivated to continue being active

Muscles become stronger

Your strength and balance improves and you feel more steady on your feet

You feel better and more confident

Top tips for physical activity in falls and frailty:

01 Being more active helps to promote confidence, prevent physical decline and gives a purpose and enjoyment to each day.

02 Exercise is often enjoyable in groups with social interaction helping with motivation, support and fun.

03 Try and build small amounts of activity into your daily routine in episodes of more than 10 minutes at a time.

04 Build up exercise gradually and start gently.

05 Exercise and being more active can help reduce your risk of falling, improve confidence and physical functioning for daily tasks.

06 Consider the use of chair based exercise programmes.

Activities

- Cycling
- Walking
- Join a motivational group

At Home

- Gardening
- Housework – doing the hoovering
- Stand during an advert break
- Avoid prolonged sitting

Travelling

- Cycling
- Walking
- Public transport

UK Chief Medical Officers' physical activity guidelines

Doing some physical exercise is good and every second counts so it's never too late to start.

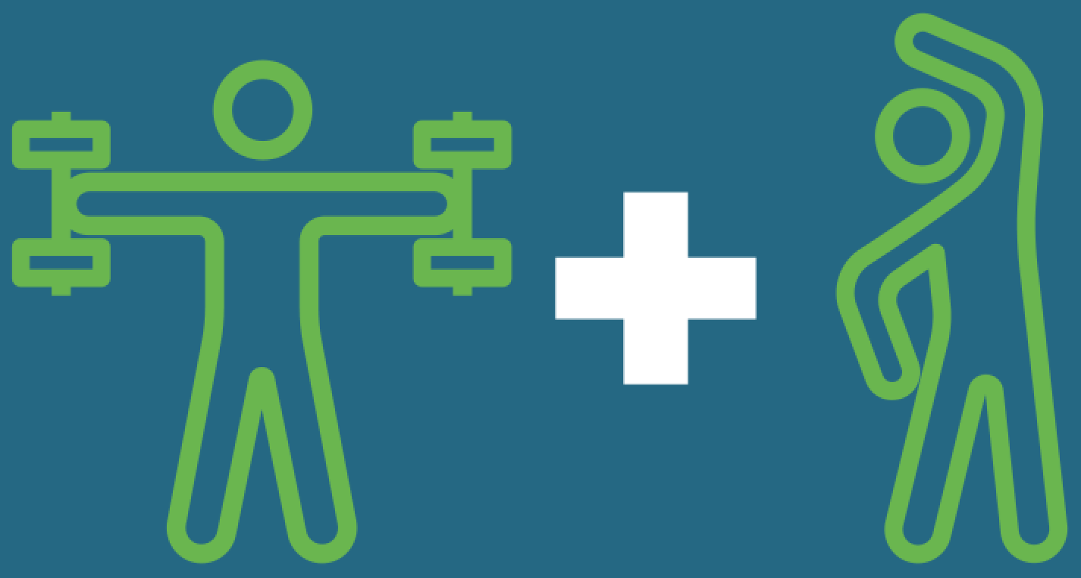

Build strength and improve balance

Keep **muscles, bones** and **joints** strong

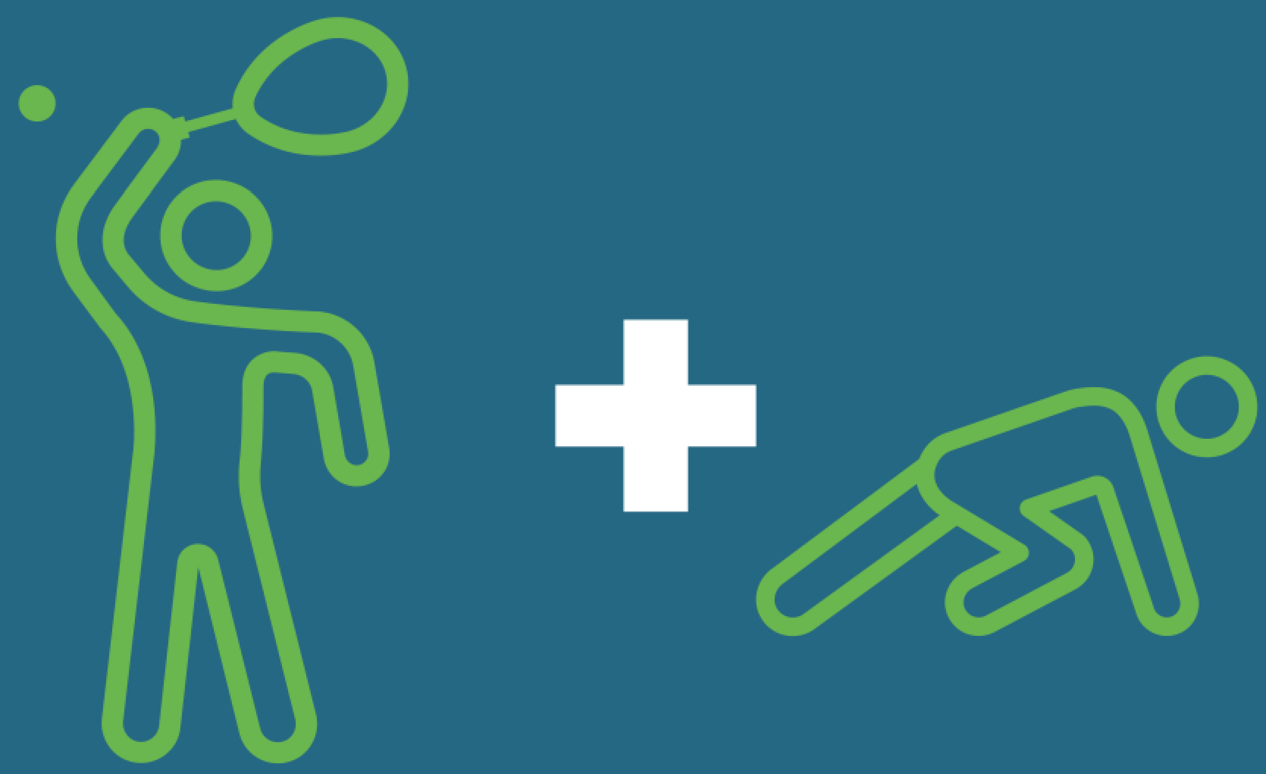

Be active

Including **moderate** and **vigorous** activities

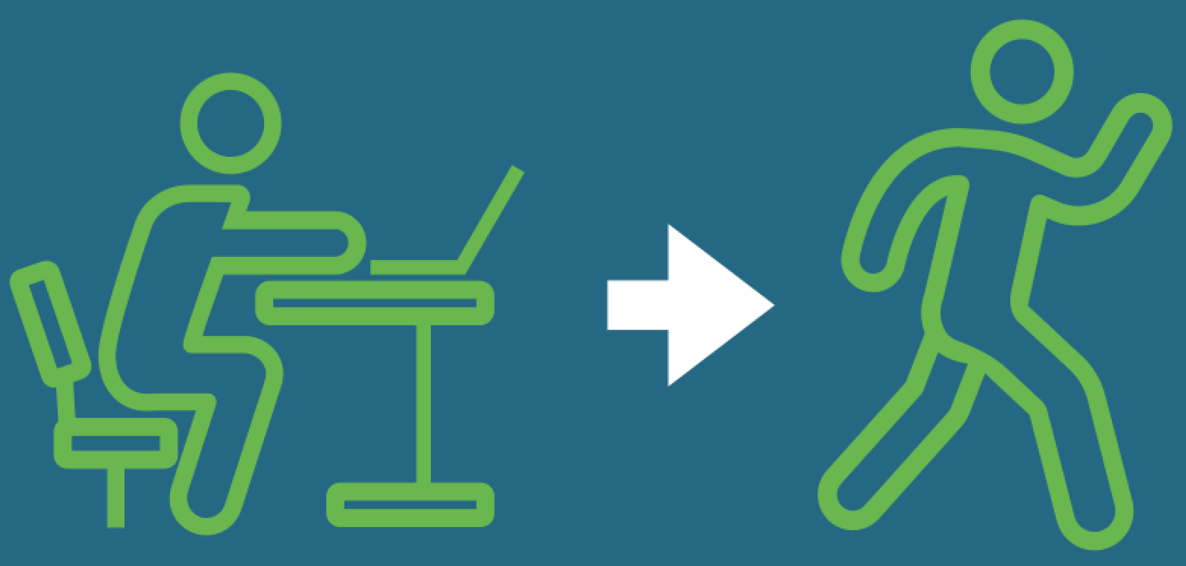

Minimise sedentary time

Break up periods of inactivity

Public Health England

Health Matters

Improve strength and balance

These activities are important across the life course for different reasons:

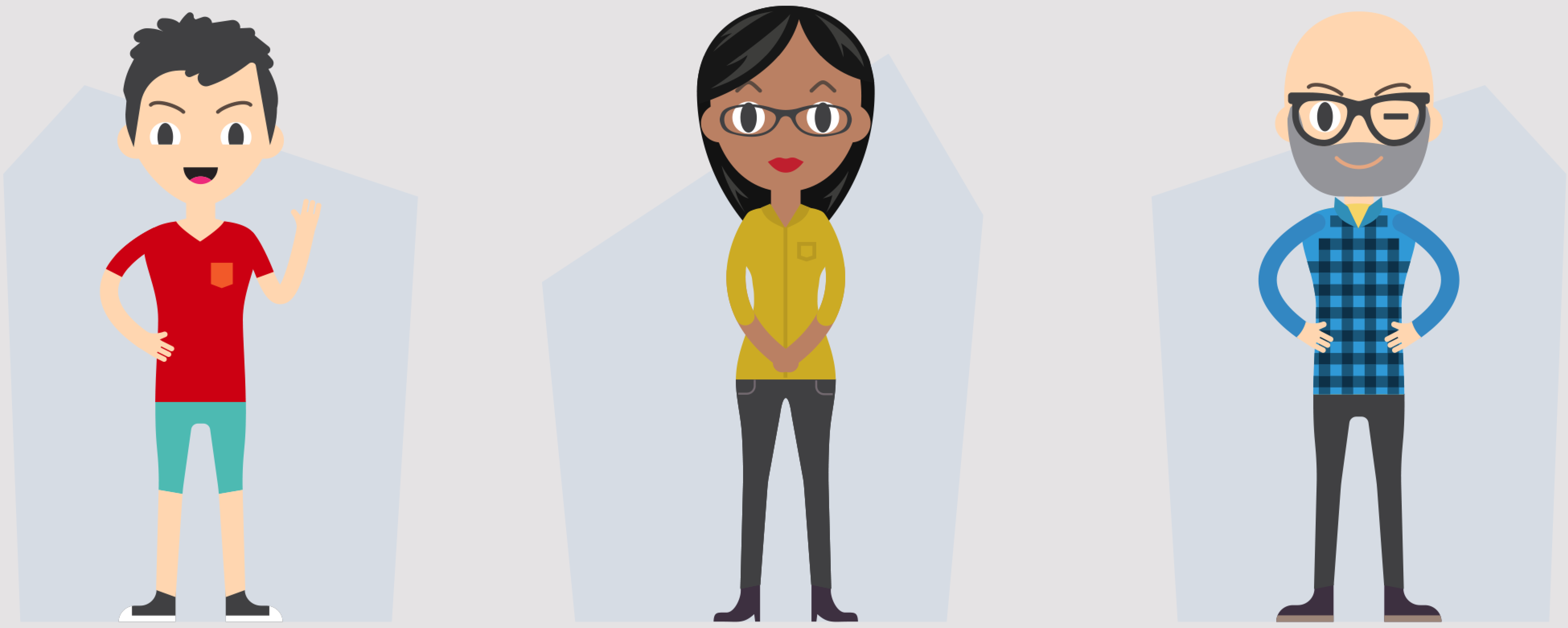

At ages 18 to 24 years

Helps to maximise bone and muscle gains

At ages 40 to 50 years

Helps to maintain strength and slows natural decline in muscle mass and bone density

At ages over 65 years

Helps to preserve strength and maintain independence

We should all aim to do strength and balance activities two to three times a week.

What counts as strengthening and balance activities

Aim to do muscle strengthening and balance activities at least two days a week

- Tai Chi
- Resistance training
- Carrying heavy shopping
- Ball games
- Racquet sports

Be active and minimise sedentary time

The UK Chief Officers' physical activity guidelines recommended that each week, adults should aim for:

150 minutes of **moderate intensity** physical activity

Or

75 minutes of **vigorous intensity** physical activity

Or

A combination of **moderate,** and **vigorous** intensity physical activity

Regular physical activity reduces your risk of

- Dementia by **30%**
- Hip fractures by up to **68%**
- Depression by up to **30%**
- Breast cancer by **20%**
- All-cause mortality by **30%**
- Cardiovascular disease by **35%**
- Type 2 diabetes by up to **40%**
- Colon cancer by **30%**

What counts as moderate intensity physical activity

Being able to talk but not sing indicates moderate intensity activity

- Brisk walking
- Cycling
- Gardening
- Dancing
- Exercise class

What counts as vigorous intensity physical activity

Having difficulty talking without pausing is a sign of vigorous activity

- Swimming/Water aerobics
- Hiking
- Running
- Active sports
- Heavy weight training

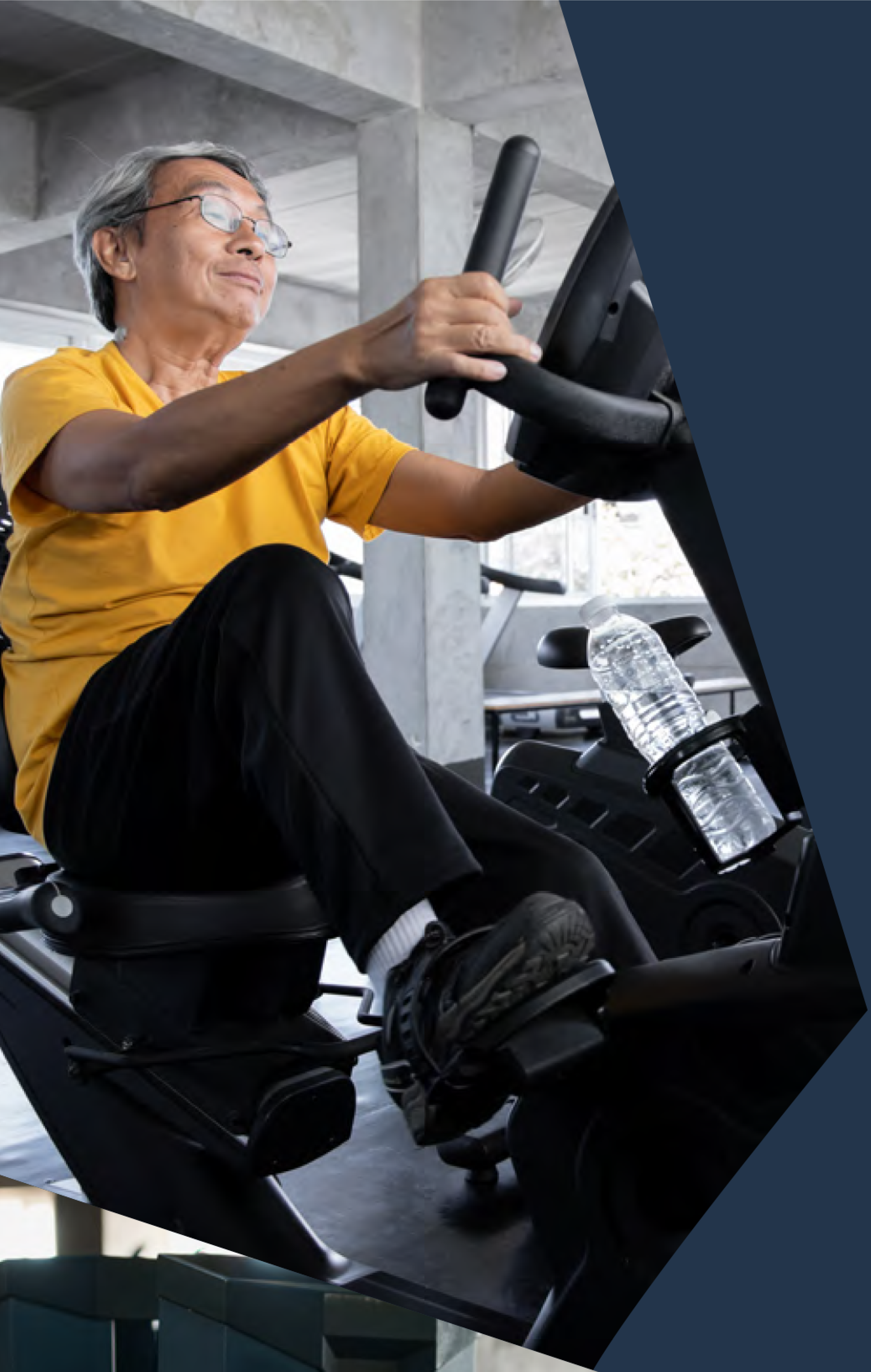

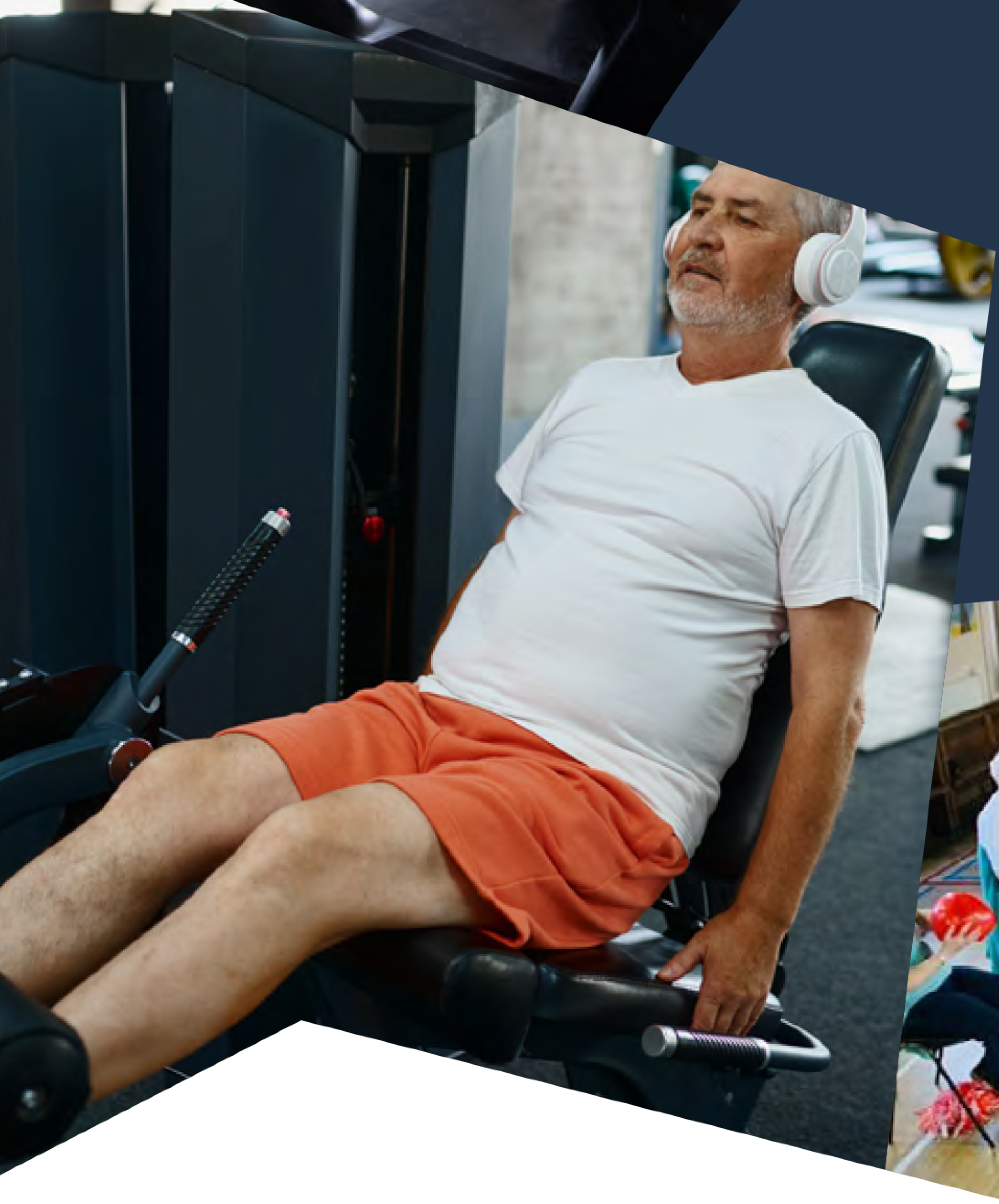

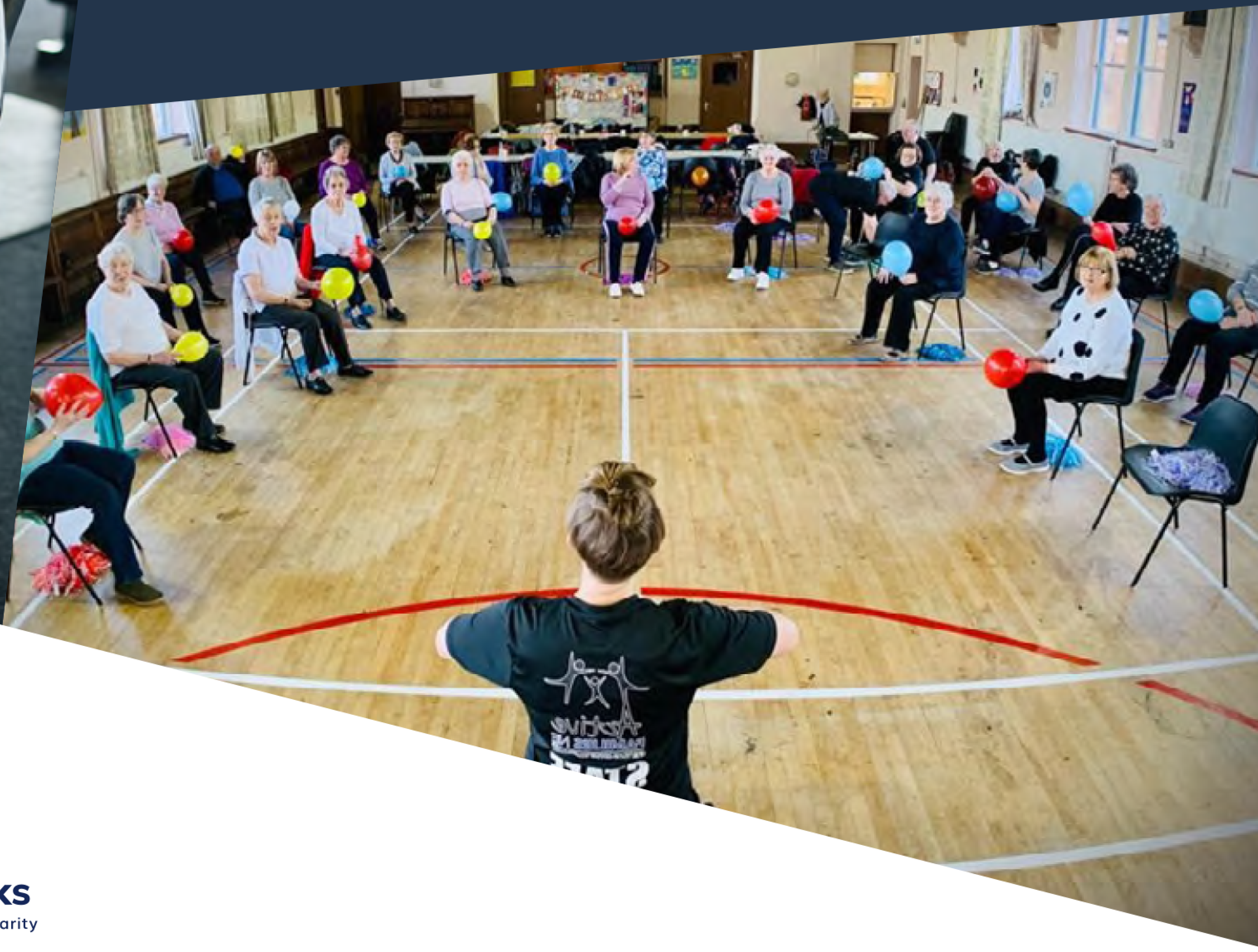

Supported by:

RISE.

Healthworks
the community health charity

GORILLA TRAINING

mindful therapies

Active FAMILIES NE

West End Family Health

BOWLS DEVELOPMENT ALLIANCE.
